# Supplementary material for: Deep-tissue two-photon brain imaging enabled by a tunable fiber-optic dispersive wave generator
Source: Sci Rep. 2025 Jul 8;15:24404. doi: 10.1038/s41598-025-08704-w (PMC12238497; doi:10.1038/s41598-025-08704-w)
Supplement: Supplementary file 1 — Supplementary Material 1 [file 41598_2025_8704_MOESM1_ESM.docx]

Deep-Tissue Two-Photon Brain Imaging Enabled by a Tunable Fiber-Optic Dispersive Wave Generator: Supplementary Information

S1. Relative Intensity Noise Transfer of the Dispersive Wave Generation

To quantify the frequency-resolved intensity noise of the dispersive wave (DW) generator, the single-sideband relative intensity noise (RIN) spectra of the driving Yb:fiber laser (YFL) and the filtered DW outputs at 880 nm, 920 nm, and 950 nm are measured using a fast photodetector and signal source analyzer, as described in the Methods section of the main manuscript.


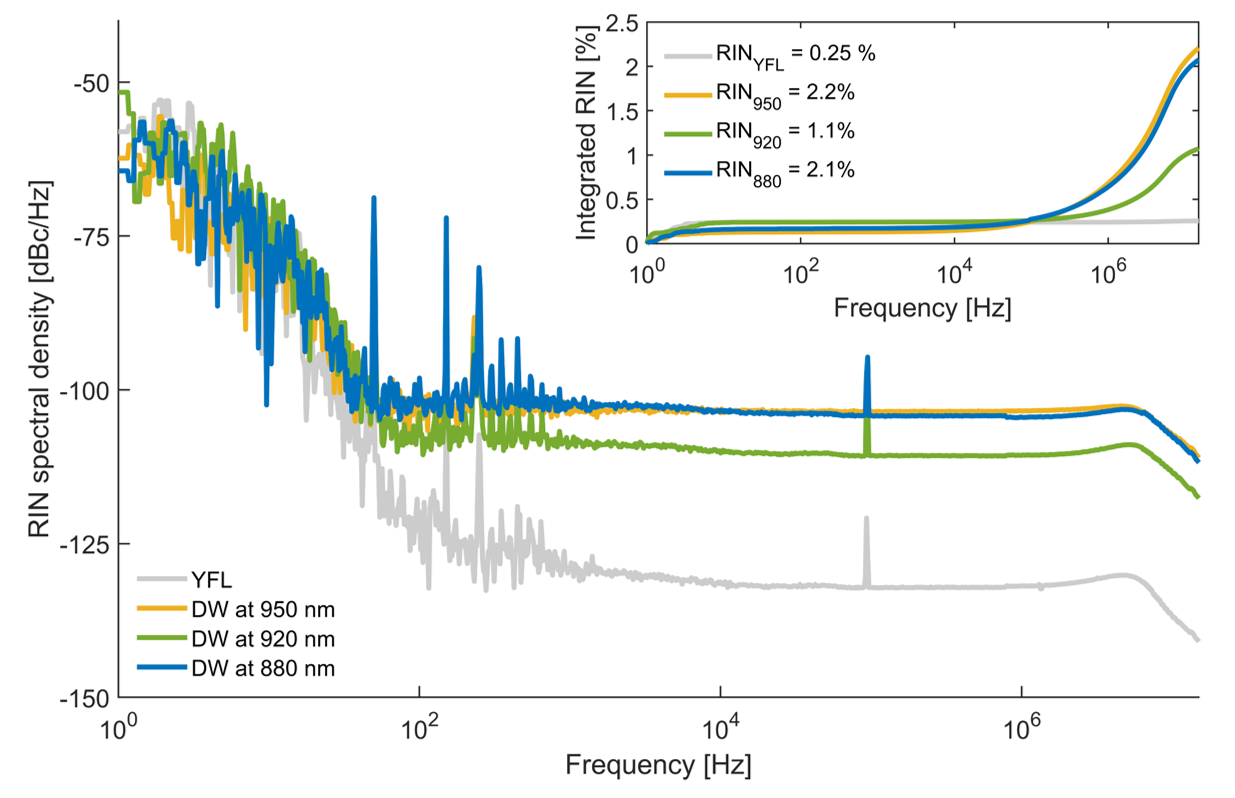


**Fig. S1:** Frequency-resolved single-sideband RIN spectra (1 Hz to 15 MHz) of the YFL (gray) and the dispersive wave (DW) outputs at 880 nm (blue), 920 nm (green), and 950 nm (yellow) center wavelength. The inset shows the corresponding integrated RIN values: ${RIN}_{YFL}=0.25\%,$ ${RIN}_{950}=2.2\%$, ${RIN}_{920}=1.1\%$ and ${RIN}_{880}=2.1\%$.

Supplementary Fig. S1 shows the single-sideband RIN spectra over a frequency range of 1 Hz to 15 MHz, along with the corresponding integrated RIN (inset). The YFL with integrated RIN of ~0.25% serves as a low-noise reference. In comparison, the DW outputs show increased RIN due to nonlinear noise transfer during soliton fission and dispersive wave emission, with broadband amplification of the RIN spectral density at frequencies above ~90 Hz. The DW at 920 nm exhibits the lowest noise among the characterized DW wavelengths (~1.1% RIN), while the DWs at 880 nm and 950 nm show integrated RIN values of ~2.1% and ~2.2%, respectively. Compared to the numerically simulated DW at 920 nm, which showed a relative peak-power fluctuation of ~0.73%, the experimentally measured RIN is slightly higher. This can be attributed to additional noise sources not included in the simulation, such as amplified spontaneous emission from the YFL, which increases the effective input noise beyond the ideal shot-noise-limited quantum fluctuations assumed in the model.

S2. SHG-Based Stability Measurement

To evaluate the pulse-to-pulse intensity stability of the DW generator under realistic conditions, the second-harmonic generation (SHG) signal produced in a 3 mm-long, type-I phase-matched BBO crystal (θ = 23°) is characterized using the 950 nm DW output. Due to its nonlinear dependence on peak power, the SHG signal provides a sensitive probe of pulse energy fluctuations and directly reflects the influence of temporal stability on two-photon imaging performance. For comparison, we also characterized the SHG stability of the driving YFL, which serves again as a low-noise reference. At both wavelengths, SHG is performed with 50 mW average input power, focused into the BBO using a 19 mm focal lens (Thorlabs AC-127-019-B) enabling ~20% conversion efficiency. To quantify the relative noise of both sources as function of time, the RMS voltage fluctuations (ΔV/V) are recorded at 0.1 Hz sampling rate over a 600-second interval using the photo-detected pulse train and a digital oscilloscope (Rohde & Schwarz HMO722). The YFL output near 1030 nm is monitored with a fast InGaAs photodetector (Coherent ET-3010), while the YFL SHG signal at 515 nm, the DW fundamental at 950 nm, and the DW SHG signal at 475 nm are detected using a fast Si-based photodetector (Thorlabs PDA10A2).


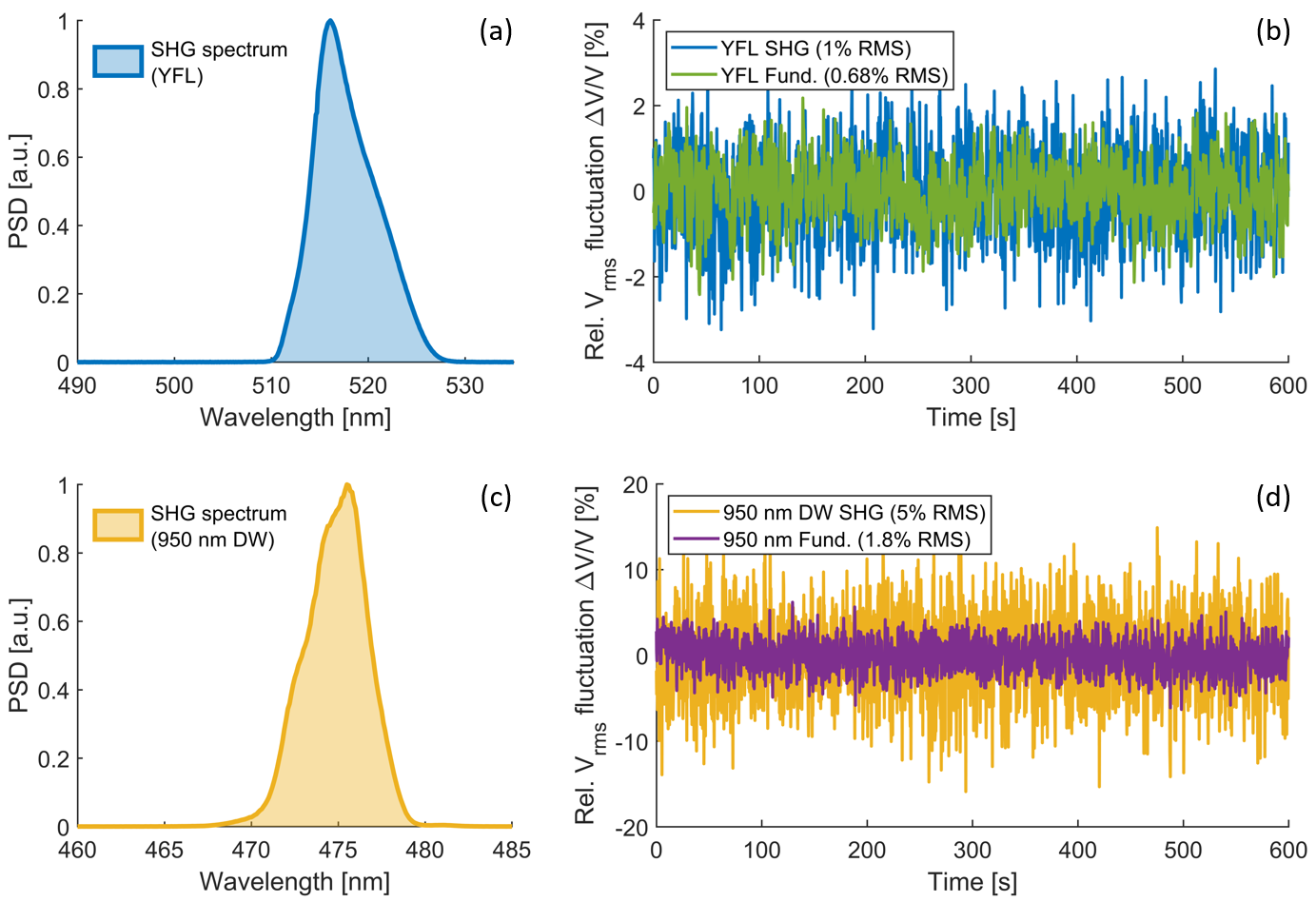


**Fig. S2:** SHG signal spectra and time-resolved stability measurements of the YFL and 950 nm output of the DW generator. (a): Measured SHG spectrum of the YFL output (~1050 nm fundamental), centered at ~515 nm. (b): Time trace of the normalized SHG signal (blue) and fundamental output (green) from the YFL, showing RMS fluctuations of 1.0% and 0.68%, respectively. (c): SHG spectrum of the 950 nm DW output, centered at ~475 nm. (d): Corresponding time traces of the DW SHG signal (yellow) and fundamental (purple), showing higher RMS fluctuations of ~5.0% and 1.8%, respectively.

As shown in Supplementary Fig S2 (a), the SHG of the YFL yields a clean spectral peak around 515 nm. The corresponding time trace of the relative RMS voltage noise $\Delta V/V$ in Fig. S1 (b) indicates relative RMS fluctuations of ~1.0% in the SHG signal and ~0.68% in the fundamental, confirming the low-noise characteristics of the source. Fig.S2 (c) shows the SHG spectrum of the 950 nm DW, centered at ~475 nm, and the corresponding time trace in Fig.S2 (d), revealing increased fluctuations with ~5% RMS in the SHG and ~1.8% in the fundamental.

S3. Imaging Stability vs. Frame Averaging

To evaluate the impact of temporal noise and averaging on image quality, we performed two-photon fluorescence imaging of fixed mouse brain tissue in the cerebellum with SYTOX Orange stained neuronal nuclei using the dispersive wave output at 950 nm center wavelength (corresponding to the DW stability experiment in S2). Images are repeatedly acquired at an imaging depth of 200 μm in a 461x461 µm field of view (FOV, 1024x1024 pixels) with 0.45 μm pixel size while varying the number of averaged frames (N = 0, 3, 6, 10, 12, 15). The average power on the sample is kept at a constant ~25 mW for all measurements. As shown in Supplementary Fig. S3, images without averaging (N = 0) display visible minor fluctuations in brightness and reduced contrast across the frame. As the number of averaged frames increases, the signal becomes progressively smoother and more uniform, with significant improvement already visible at N = 3. From N ≥ 10, the image quality appears nearly saturated, confirming that the system maintains high stability under short integration times and performs robustly even with minimal averaging. These results highlight the DW generators potential also for time-resolved or high-speed imaging applications, particularly when combined with additional standard noise suppression techniques based on active feedback control or passive pulse stabilization.


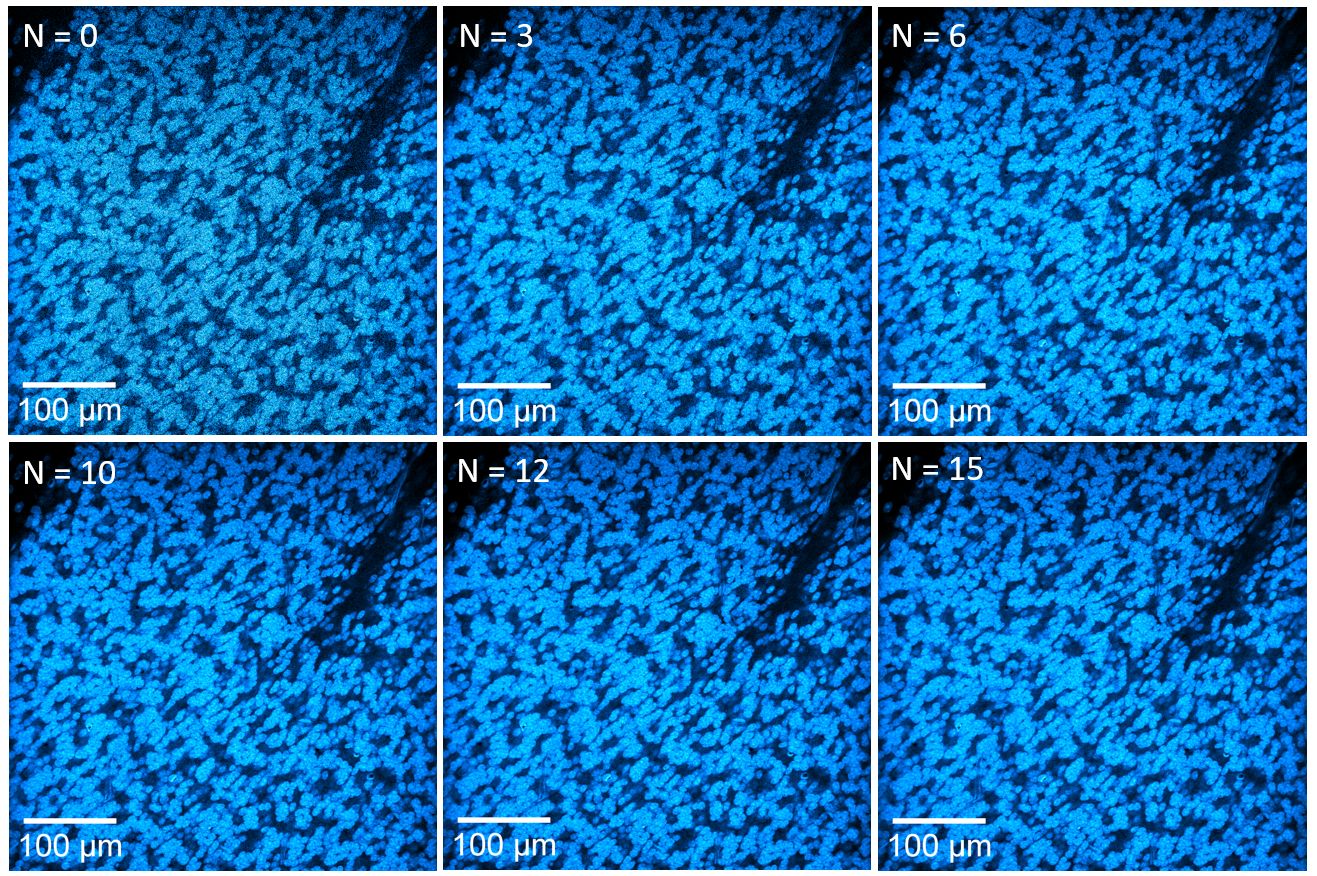


**Fig. S3:** Effect of frame averaging on image quality in two-photon fluorescence imaging. SYTOX Orange-labeled neuronal nuclei in fixed mouse cerebellum were imaged at 950 nm excitation with increasing numbers of averaged frames: N = 0, 3, 6, 10, 12, 15. All images were acquired under identical conditions. Increased averaging suppresses temporal noise and enhances signal consistency, with substantial improvements already evident at N = 3. Scale bar: 100 µm.
